# Watching without walls: a scoping review of electronic sitters for preventing falls and fall-related injuries among hospitalized patients

**DOI:** 10.3389/fdgth.2026.1969777

**Published:** 2026-10-29

**Authors:** Alexis Harerimana, Gugu Mchunu

**Affiliations:** 1 Faculty of Health Sciences, Durban University of Technology, Durban, KwaZulu-Natal, South Africa; 2 College of Healthcare Sciences, James Cook University, Townsville, QLD, Australia

**Keywords:** electronic sitters, fall prevention, hospitalized patients, patient safety, telesitters, video monitoring, virtual sitters

## Abstract

**Background:**

Falls are among the most frequent and costly adverse events in hospitals, and conventional one-to-one sitters are resource-intensive with uncertain effectiveness. Electronic sitters offer a scalable alternative, but their characteristics, outcomes, and implementation determinants remain poorly mapped. This scoping review synthesizes the literature on electronic sitters for preventing falls and fall-related injuries among hospitalized patients.

**Methods:**

This scoping review followed established methodological and reporting guidelines. Five databases (CINAHL, Emcare, MEDLINE, Scopus, and Web of Science) and manual searches were conducted to identify studies published between January 2016 and July 2026, using the Population–Concept–Context framework to determine eligibility. Of 1,806 records identified, 25 studies met the inclusion criteria and were analyzed thematically.

**Results:**

Electronic sitter technology platforms included Ocuvera, AvaSure systems, SMART AI Patient Sitter, Verso Vision, Oxehealth, Artisight-enabled virtual patient observation, Virtual Nurse, Epic Monitor systems, Uniden baby monitors, and continuous or remote video monitoring systems. Most systems used standard cameras with continuous, centralized, human-led observation; a minority incorporated AI-assisted detection. Monitoring ratios ranged from 6:1 to 15:1, with 11:1–15:1 reported most frequently, and one system's maximum technical capacity reaching 16:1. Among 22 studies reporting falls, significant reductions ranged from 25.1% to 79%, whereas injurious-fall reductions were significant in only four of ten studies. AI-augmented systems achieved 97.4%–98.4% detection sensitivity, and sitter hours declined by 10%–91%, with projected savings up to US$3.2 million. Facilitators included multidisciplinary governance, structured training, and eligibility algorithms integrated into electronic health records, while barriers included limited device supply, staff resistance, and technological limitations.

**Conclusion:**

Electronic sitters represent a promising adjunct to conventional fall-prevention practice, offering potential—though inconsistently demonstrated—resource benefits rather than a clear cost-effectiveness advantage. Deployment must address existing implementation challenges and proceed safely and ethically.

## 1 Background

Inpatient falls are among the most frequent, harmful, and preventable patient safety events in acute care (1, 2). Globally, falls are the second leading cause of fatal unintentional injury, causing an estimated 684,000 deaths annually (3). Without intervention, fall-related injuries are projected to double by 2030 (4). In hospitals, falls occur at a rate of 3–5 per 1,000 patient-days (5, 6), and these figures are markedly higher in psychogeriatric units (17–67 falls per 1,000 occupied bed-days) (6). Beyond immediate injury, inpatient falls lead to substantial secondary consequences, including elevated mortality risk among older adult fallers compared with non-fallers (5). Hospitalization costs approximately double for patients who fall (7), and remote patient monitoring (RPM) literature consistently identifies falls as a major driver of downstream healthcare utilization (8). Conventional monitoring, reliant on intermittent nursing assessments, creates surveillance gaps, especially during understaffed periods or at night when falls and clinical deterioration often go undetected until after the event (7, 9). In-person constant observation (bedside sitters) for the highest-risk patients has been documented; however, the evidence supporting traditional sitters is weak. A systematic review by Greeley et al. (10) found only very-low-certainty evidence that one-to-one sitters reduce falls, despite substantial staffing and financial costs. The preventability of falls has spurred interest in electronic sitters—also known as e-sitters, virtual sitters, continuous or remote video monitoring systems (CVM/RVM), virtual observation, or remote patient monitoring (8, 9, 11–18)—as scalable, technology-enabled strategies to provide continuous surveillance for high-risk patients.

Electronic sitting usually means replacing a traditional 1:1 in-person sitter with one remote observer who watches multiple patients by video or sensor-based systems. Trained technicians monitor many patients concurrently via live, non-recording camera feeds, with real-time two-way audio and structured escalation protocols (15, 19). The literature does not support one universal ratio. Ratios vary by patient acuity, technology, and staffing model, but the most commonly reported remote safety observation range is 8:1–12:1 patients per observer, with some artificial intelligence (AI)-augmented programs reporting up to 16:1 (11, 15, 19, 20). Electronic sitters encompass a spectrum of technologies within the rapidly expanding Internet of Medical Things (IoMT), valued at US$230.69 billion globally in 2024 (21). The dominant model is human-staffed CVM/RVM, deployed through platforms such as AvaSure/AvaSys TeleSitter (11, 22, 23), Ocuvera Automated Video Monitoring System (AVMS) (24, 25), and Epic Monitor (13). In this model, a centralized monitor technician observes live feeds and intervenes verbally before unsafe patient movement. A rapidly growing category includes AI- and computer-vision-augmented systems that algorithmically detect bed-exit risk and issue predictive alerts without continuous human observation. Examples include SMART AI Patient Sitter (26), Verso Vision (27), Oxehealth (28), and Artisight (29). Deep learning architectures in these systems achieve up to 93% accuracy in fall detection evaluations (5). Mateus et al. (30) argue that responsible AI-driven monitoring requires explicit ethical frameworks addressing consent, data security, and autonomy. Consumer-grade portable cameras are also used in resource-limited settings to extend monitoring without centralized infrastructure (31).

Empirical evidence increasingly supports electronic sitters’ potential to reduce inpatient falls and sitter costs. Greeley et al. (10) reported moderate-certainty evidence that video monitoring reduced sitter utilization while either reducing or not affecting fall rates. System-wide implementations of electronic sitters demonstrated scalability, including deployments across 15 rural critical-access hospitals and 13-hospital integrated networks (24, 25, 32). Significant reductions in sitter hours and cost savings have been documented alongside fall reductions in programs using both human-staffed and AI-augmented platforms (11, 23, 29, 33). AI-augmented video review also shows promise for secondary prevention, and Bayen et al. (34) found that algorithmic review of fall events in a dementia managed-care setting enabled the identification of cognitive-behavioral and environmental precursors in high-risk multi-fallers, supporting targeted intervention. International implementations in Malaysia (26), Italy (27), Australia (31), and the United Kingdom (UK) (28) broaden the evidence base. The broader RPM literature mirrors these findings: Tan et al. (8), in a systematic review of 29 RPM interventions, found consistent positive trends in patient safety and resource utilization, though with less consistent impact on quality-of-life outcomes.

However, the promise of electronic sitters must be balanced against documented challenges. Staff acceptance varies from strong endorsement to a preference for in-person sitters (12, 35–37), with adoption differing substantially between units within the same program (38). Device supply often falls short of eligible patient demand, concentrating fall risk among unmonitored patients rather than eliminating it (15, 18, 24). Privacy, dignity, and informed consent present ethical and governance challenges: camera-based monitoring in hospitals raises legitimate concerns about patient rights, particularly for cognitively impaired patients (39). Remote monitoring programs in the context of telerehabilitation (40), COVID-19 home monitoring (16, 41), and early-discharge programs with remote surveillance (42) reinforce both scalability and implementation complexity.

Video-based observation has an established role in clinical performance and safety assessment in high-risk environments such as emergency departments and operating rooms (43). AI-based platforms, such as Software as a Medical Device (SaMD), should meet clinical performance, transparency, and surveillance requirements for electronic sitter technology (44–46). Successful electronic sitter interventions depend critically on organizational infrastructure rather than camera choice. Clinical nursing leadership, multidisciplinary governance, structured training, and electronic health record (EHR)-embedded eligibility algorithms are consistent enablers (15, 29, 47, 48), while formal change-management frameworks—Plan-Do-Study-Act (PDSA) cycles (13), the Awareness, Desire, Knowledge, Ability, and Reinforcement (ADKAR) model (15), and the Concerns-Based Adoption Model (38)—have structured adoption in successful programs.

In light of the critical role of electronic patient surveillance and prevention of falls and related injuries, and given the heterogeneity of technologies, settings, populations, and study designs, a scoping review was the most appropriate methodology to map the existing literature about the use of electronic sitters for preventing falls and fall-related injuries among hospitalized patients.

## 2 Methods

This scoping review adhered to the methodological framework for scoping reviews, initially proposed by Arksey and O'Malley (49) and subsequently refined by Levac et al. (50). This framework consists of five essential stages: identifying the research question, identifying relevant studies, selecting studies, charting the data, and collating, summarizing, and reporting the results. Arksey and O'Malley (49) also suggested an optional sixth stage, stakeholder consultation, which was not undertaken for this review. Therefore, the findings presented reflect solely the published literature and do not incorporate contextualization from stakeholder perspectives. The review's reporting followed the Preferred Reporting Items for Systematic Reviews and Meta-Analyses Extension for Scoping Reviews (PRISMA-ScR) (51).

### 2.1 Stage 1: identifying the research question

This review was primarily designed to address the overarching research question: What is known from the existing literature regarding the utilization of electronic sitters for the prevention of falls and fall-related injuries among hospitalized patients?

To facilitate a comprehensive exploration of this multifaceted topic, the review was further guided by six complementary research sub-questions:

1. What types of electronic sitter technologies have been used to prevent falls and related injuries among hospitalized patients?
2. How have electronic sitter interventions been implemented to prevent falls and related injuries among hospitalized patients?
3. What outcomes have been reported following the use of electronic sitters among hospitalized patients?
4. How effective are electronic sitters in reducing patient falls and fall-related injuries among hospitalized patients?
5. What are the reported barriers associated with the use of electronic sitters in hospital settings?
6. What factors facilitate the implementation of electronic sitters in hospital settings?

Consistent with the methodological guidance provided by Levac et al. (50), the primary and sub-research questions, along with the Population–Concept–Context (PCC) criteria, underwent iterative refinement by the review team as their familiarity with the emergent literature deepened. This iterative approach is a defining characteristic of the Levac et al. (50) framework, ensuring that the review question remains responsive and optimally aligned with the evolving evidence base.

### 2.2 Stage 2: identifying relevant studies

A systematic search was conducted across the Cumulative Index to Nursing and Allied Health Literature (CINAHL), Emcare, Medical Literature Analysis and Retrieval System Online (MEDLINE), Scopus, and Web of Science. Manual searches supplemented this using Google and Google Scholar. Inclusion criteria were delineated using the PCC framework. The Population domain included hospitalized, inpatient, and acute-care patients of all ages requiring observation for fall prevention. The Concept domain referred to electronic sitter technologies and related interventions, such as electronic sitters, e-sitters, esitters, virtual sitters, telesitters, tele-sitters, remote patient monitoring systems, video monitoring, continuous video monitoring, virtual observation systems, and remote observation technologies. The Context domain included hospitals, hospital settings, inpatient units, acute care settings, medical wards, and surgical wards.

The search strategy combined the following terms using Boolean operators: hospitalized patients, electronic sitters, hospital settings, and falls. Searches were explicitly limited to studies published in English from 2016 to 2026. Furthermore, database searches were conducted from May 2026 to July 2026, with a final update search completed in July 2026. Subject headings were exploded where applicable (denoted by “exp” in MEDLINE and Emcare and by the “+” symbol in CINAHL) to retrieve narrower terms. For example, exploding broad headings for falls, hospitals, and telemedicine captured their narrower terms. This approach ensured comprehensive coverage and improved reproducibility. In Scopus and Web of Science, which lack hierarchical controlled vocabularies, the same concepts were searched as free-text terms in the title, abstract, and keyword fields (TITLE-ABS-KEY in Scopus and Topic search in Web of Science). Consequently, records describing electronic sitter technologies using terminology not captured by the free-text terms (e.g., proprietary product names) may not have been retrieved in these two databases, which is acknowledged as a limitation.

Google and Google Scholar were used to identify gray literature and unindexed records, including preprints, conference abstracts, and quality-improvement reports. Neither platform supports controlled vocabularies or term expansion, and Google Scholar limits query length. Therefore, simplified keyword combinations of the main concepts (e.g., “virtual sitter” or “telesitter” combined with “fall” and “hospital”) were employed. Because results are ranked by relevance and cannot be exported comprehensively, the first 100 results from each Google Scholar search and the first 10 pages from each Google search were screened. This approach is commonly used to make gray literature searching feasible, as suggested by Haddaway et al. (52). The search strategy was customized to each database, and the following is an example of a MEDLINE Ovid search string:

**Search 1 (Population)**

1. exp Inpatients/ or exp Hospitalization/ or “hospitali?ed patient*”.mp. or inpatient*.mp. or “acute care patient*”.mp. or patient*.mp. or “admitted patient*”.mp.

**Search 2 (Concept)**

2. (“electronic sitter*” or e-sitter* or esitter* or “virtual sitter*” or telesitter* or tele-sitter* or “remote patient monitoring” or “video monitoring” or “continuous video monitoring” or “virtual observation” or “video observation” or “remote observation”).mp.

**Search 3 (Context)**

3. exp Hospitals/ or exp Hospital Units/ or exp Emergency Service, Hospital/ or exp Intensive Care Units/ or hospital*.mp. or “hospital setting*”.mp. or “inpatient unit*”.mp. or “acute care setting*”.mp. or ward*.mp. or “medical ward*”.mp. or “surgical ward*”.mp. or “emergency department*”.mp. or “intensive care unit*”.mp.

**Search 4 (Outcome)**

4. exp Accidental Falls/ or exp Fall Prevention/ or exp Patient Safety/ or “patient fall*”.mp. or “inpatient fall*”.mp. or “hospital fall*”.mp. or “fall prevention”.mp. or “fall reduction”.mp. or “fall-related injur*”.mp. or “injury prevention”.mp. or “patient safety”.mp.

**Search 5 (Combination of search blocks)**

5. 1 AND 2 AND 3 AND 4

### 2.3 Stage 3: study selection

Study selection was conducted in two distinct phases, managed manually using a shared reference-management library (EndNote, Version 21) and a spreadsheet. In the initial phase, titles and abstracts were independently screened by two reviewers against the eligibility criteria below. Studies were included if they (a) involved hospitalized patients; (b) evaluated an electronic sitter technology for patient monitoring; (c) were conducted in any hospital setting; (d) reported at least one fall or fall-related injury outcome; (e) were primary studies of any design, quality improvement projects, or program evaluations reporting original data, including gray literature such as doctoral projects and conference proceedings; and (f) were published in English between January 2016 and July 2026. Studies were excluded if they (a) were conducted outside hospital settings; (b) evaluated monitoring without electronic or remote patient observation; (c) reported neither a fall or fall-related injury outcome nor any other implementation outcome related to electronic sitters used for fall prevention; (d) were editorials, commentaries, letters, study protocols, or reviews, or were conference abstracts without an available full text; (e) were published in a language other than English; or (f) were published outside the January 2016–July 2026 study period.

In the second phase, full texts of all potentially eligible records were independently assessed by two reviewers, and all disagreements regarding eligibility were resolved through structured discussion between the two reviewers to consensus; no third reviewer was involved in adjudication. Reasons for exclusion at the full-text stage were meticulously recorded. The entirety of the study selection process is transparently reported using a PRISMA-ScR flow diagram, which systematically documents the number of records identified, duplicates removed, records screened, records excluded at the title/abstract stage, full texts assessed, full texts excluded with specific reasons, and the final number of studies ultimately included in the review. Study selection was directed by the review question, and the methodological rigor of eligible studies was appraised using the Mixed Methods Appraisal Tool (MMAT), version 2018 (53). The MMAT was selected because it accommodates a broad spectrum of research designs, namely qualitative studies, randomized controlled trials, non-randomized quantitative studies, quantitative descriptive studies, and mixed-methods studies (53). Each study was initially assigned to one of the five MMAT design categories and subsequently scrutinized against the criteria specific to that category, with each criterion rated as “Yes,” “No,” or “Can’t tell.” No study was excluded on the basis of its appraisal outcome; rather, the MMAT ratings shaped the interpretation of the findings by highlighting the methodological strengths and weaknesses of the evidence base for electronic sitters for preventing falls and fall-related injuries among hospitalized patients. Of 1,806 records identified, 25 studies met inclusion criteria and underwent thematic analysis (Figure 1).

**Figure 1:**
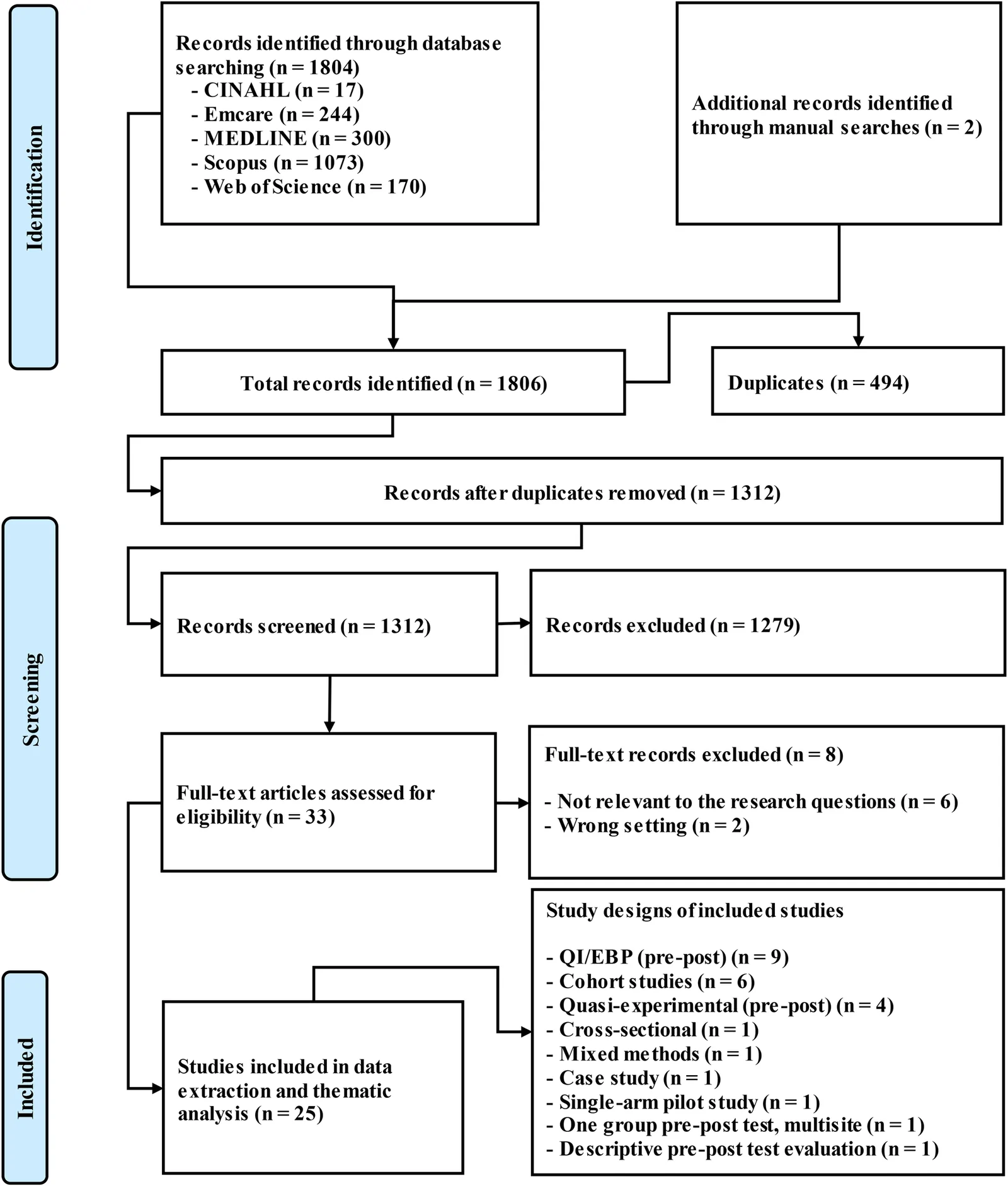
PRISMA flow diagram.

### 2.4 Stage 4: charting the data

The review team collaboratively developed a standardized data charting form to systematically extract and organize pertinent information from the included studies. This form underwent independent pilot testing by two reviewers on a purposive sample of five included studies before finalization. Feedback garnered from the pilot phase informed iterative refinements to the form, thereby ensuring that all relevant variables were comprehensively captured and that the form was clear, unambiguous, and feasible to use. This iterative refinement is a distinguishing characteristic of the Levac et al. (50) approach, setting it apart from more rigid systematic review protocols. The finalized charting form systematically captured the following variables: author(s), publication year, country of origin, study title, setting characteristics, population, sample size, study design (Table 1), taxonomy of electronic sitter technologies and interventions (Table 2), outcomes of electronic sitter interventions (Table 3), facilitators (Table 4), and barriers (Table 5).

**Table 1:** Characteristics of included studies.

Author and year	Country	Setting	Population	Sample size	Study design
Allen et al. (47), 2023	USA	2 medical-surgical units, acute care hospital	Nursing staff; high-fall-risk inpatients	*n* = 51, 37, and 30 nurses (3 survey waves); patients (NS)	Quasi-experimental pre-post
Baez and Karl (35), 2022	USA	Medically complex unit, specialty/rehabilitation hospital	High-fall-risk inpatients; nursing/PCT staff	*n* = 37 staff (25 nurses, 11 PCTs, 1 unidentified staff); patients (NS)	QI project; pre-post
Charles (13), 2023	USA	25-bed medical-surgical unit, community hospital	High-fall-risk adult patients	*n* = 64 patients (22 pre-, 42 post-intervention)	QI project (PDSA cycles); pre-post
Cook et al. (22), 2020	USA	Level 1 trauma center ED	Adult ED patients at fall risk	>80,000 patients screened (KINDER 1)	QI project; pre-post
Cournan et al. (19), 2018	USA	115-bed rehabilitation hospital	Fall-risk inpatients (brain injury unit)	15 monitored beds (of 115); 3,641 monitored vs. 5,788 non-monitored patient-days	Sequential cohort (pre-post)
Danial et al. (26), 2025	Malaysia	Acute stroke unit, public hospital	Hospitalized stroke patients	*n* = 30 patients	Prospective single-arm pilot
Davis and Carter-Templeton (55), 2021	USA	30-bed orthopedic unit, community medical center	High-fall-risk orthopedic inpatients; staff	*n* = 34 patients; 30 staff	QI project; pre-post
Ergai et al. (36), 2024	USA	41 units, 4-hospital health system	High-fall-risk inpatients; nursing staff	*n* = 106 nurses; patients (NS)	Quasi-experimental pre-post
Gervasi et al. (27), 2025	Italy	Medicine and oncology wards, general hospital	Adult inpatients	*n* = 362 patients	Retrospective observational cohort
Hebb et al. (12), 2021	USA	Medical-surgical units, academic hospital	Nurses, PCTs, nursing assistants	*n* = 55 staff (31 nurses)	Cross-sectional descriptive survey
Hogan Quigley et al. (18), 2022	USA	244-bed Magnet hospital, hospital-wide (COVID-19)	Adult inpatients at fall risk	*n* = 93 patients	QI project; pre-post
Jones et al. (24), 2021	USA	15 small rural/critical access hospitals	High-fall-risk inpatients	*n* = 372 analyzed (221 baseline, 151 intervention)	One-group pretest-posttest, multi-site
Kalafus et al. (11), 2025	USA	137-bed long-term acute care hospital	LTACH patients (altered mental status, high acuity)	*n* = 448 patients	Prospective cohort with historical comparator
Kramer et al. (25), 2020	USA	17 sites, mixed hospital types	Fall-risk inpatients	*n* = 2,473 monitored patients (17 sites)	Case study; pooled pre-post
Purvis et al. (38), 2018	USA	8 adult inpatient units, academic medical center	Adults requiring constant observation	*n* = 167 patients	QI project (Concerns-Based Adoption Model)
Sacks and Wong (15), 2025	USA	4 campuses, academic health system	Inpatients requiring constant observation	27 VCO cameras; mean 15.96–25.58 patients/month	QI project (ADKAR model); pre-post
Sand-Jecklin et al. (33), 2016	USA	4 medical-surgical units, academic medical center	High-fall-risk inpatients	*n* = 1,508 monitoring cases	Quasi-experimental pre-post
Sand-Jecklin et al. (37), 2019	USA	4 medical-surgical units, academic medical center	Nursing staff, monitor technicians, patients/families	*n* = 130 staff; 7 technicians; 52 patients/families	Mixed-methods, descriptive process evaluation
Sosa et al. (56), 2024	USA	550-bed academic tertiary care hospital	High-fall-risk adult inpatients	*n* = 9,034 patients	Retrospective, historically controlled cohort
Spano-Szekely et al. (54), 2019	USA	Medical-surgical units, 245-bed community hospital	Adult fall-risk inpatients	NS (hospital-wide; FY2014 vs. FY2016–2017)	EBP improvement (PDSA); pre-post
Tyransky et al. (29), 2025	USA	53-bed rehabilitation/brain injury unit	Rehabilitation inpatients, nurses	Average of 44 nursing staff (range 24–63) completed 7 anonymous surveys; patients (NS)	QI project (PDSA); single-center pre-post
Votruba et al. (23), 2016	USA	3 adult inpatient units, 350-bed hospital	Adult inpatients (algorithm-selected)	*n* = 828 patients	Prospective descriptive pre-post
Woltsche et al. (31), 2022	Australia	3 wards across 2 tertiary hospitals	High-fall-risk inpatients; nursing staff	494 monitoring episodes (nights); 31 night-shift staff surveyed	Cohort study; prospective pre-post
Wright and Singh (28), 2022	UK	2 dementia inpatient wards	Dementia inpatients; staff	∼11 patients per ward nightly; 12 staff surveyed	Cohort study; pre-post
Zimbro et al. (32), 2024	USA	13-hospital health system (2 RPM, 11 non-RPM)	Adult inpatients assessed for fall risk	174,511 pre- and 180,475 post-intervention visits (RPM: 22,343 and 21,504; non-RPM: 152,168 and 158,917)	Quasi-experimental, controlled pre-post

AI, artificial intelligence; ED, emergency department; EHR, electronic health record; JHFRAT, Johns Hopkins Fall Risk Assessment Tool; LTACH, long-term acute care hospital; NS, not specified; PCT, patient care technician; PDSA, Plan-Do-Study-Act; QI, quality improvement; RPM, remote patient monitoring.

**Table 2:** Technology taxonomy of electronic sitter interventions.

Technology taxonomy	Studies (n)	Descriptions of features	Country	Studies
Technology or vendor				
Ocuvera AVMS	(*n* = 3)	Kinect depth cameras predict bed exits; de-identified silhouettes protect privacy.	USA	(24, 25, 47)
AvaSure Systems (AvaSys TeleSitter; AvaSure Guardian)	(*n* = 3)	Non-recording cameras with two-way audio; tiered escalation to second responder.	USA	(11, 22, 23)
SMART AI Patient Sitter	(*n* = 1)	AI optical sensor detects bed exits and falls; blurred images protect privacy.	Malaysia	(26)
Verso Vision	(*n* = 1)	AI computer vision with smartphone alerts for bed exits and falls.	Italy	(27)
Oxehealth	(*n* = 1)	Contact-free infrared sensor tracks vital signs; no continuous video feed.	UK	(28)
Artisight-enabled VPO + Virtual Nurse	(*n* = 1)	AI-augmented in-room cameras with fall-risk indicators and Virtual Nurse.	USA	(29)
Epic Monitor (Virtual Sitter)	(*n* = 1)	EHR-integrated cameras with two-way audio; one sitter observes up to 10 patients.	USA	(13)
Uniden Baby Monitors	(*n* = 1)	Low-cost baby monitors with motion/sound alerts; overnight use only.	Australia	(31)
Technology or Vendor for CVM/RVM (NS)	(*n* = 13)	Live-feed cameras with two-way audio; technicians monitor 6–15 patients.	USA	(12, 15, 18, 19, 32, 33, 35–38, 54–56)
Sensing modality				
Depth sensor	(*n* = 3)	Kinect depth cameras generate de-identified silhouettes for bed-exit prediction.	USA	(24, 25, 47)
Multimodal (optical + thermal)	(*n* = 1)	Fisheye and thermal cameras detect bed exits, falls, staff presence.	Malaysia	(26)
Infrared/other sensor (non-displaying computer vision)	(*n* = 1)	Infrared video sensor tracks vital signs without continuous display.	UK	(28)
Standard camera/video	(*n* = 20)	Live cameras with two-way audio (12, 29, 33, 36, 37, 55); infrared night vision (23, 38), AI-processed (27).	USA, Italy, Australia	(11–13, 15, 18, 19, 22, 23, 27, 29, 31–33, 35–38, 54–56)
Continuous human observation vs. event-triggered review				
Continuous human observation	(*n* = 16)	Technicians continuously watch live video and intervene via two-way audio.	USA	(11–13, 15, 18, 19, 22, 23, 32, 33, 36–38, 54–56)
Event-triggered/intermittent review (non-automated)	(*n* = 1)	Unit nurses checked video intermittently; no automated trigger.	USA	(35)
Automated alert followed by human review	(*n* = 8)	Sensor-triggered alerts, including AI and motion/sound systems, prompt staff review.	USA, Malaysia, Italy, Australia, UK	(24–29, 31, 47)
AI-assisted vs. automated detection				
AI-assisted	(*n* = 7)	Machine-learning or neural-network detection of bed exits and falls.	USA, Malaysia, Italy, UK	(24–29, 47)
Automated/rule-based (non-AI)	(*n* = 1)	Motion/sound-triggered alerts; no AI detection.	Australia	(31)
Human-only monitoring	(*n* = 17)	Detection relies entirely on human observation.	USA	(11–13, 15, 18, 19, 22, 23, 32, 33, 35–38, 54–56)
Centralized vs unit-based monitoring				
Centralized monitoring	(*n* = 16)	Monitoring consolidated in one room or off-site center.	USA, Malaysia	(11–13, 15, 18, 19, 22, 23, 26, 29, 32, 33, 36–38, 56)
Unit/ward-based monitoring	(*n* = 4)	Alerts go directly to single-unit staff; no central hub.	USA, UK	(28, 35, 47, 55)
Decentralized monitoring	(*n* = 4)	Alerts pushed to staff mobile devices; no fixed location.	USA, Italy, Australia	(24, 25, 27, 31)
Dedicated observer vs existing bedside staff				
Dedicated observer/monitoring staff	(*n* = 14)	Dedicated monitoring role separate from bedside nursing.	USA	(11–13, 15, 18, 19, 22, 23, 29, 32, 33, 36, 37, 55)
Existing bedside nurses/clinical staff	(*n* = 8)	Bedside nurses or ward staff handle alerts.	USA, Malaysia, Italy, Australia, UK	(24–28, 31, 35, 47)
Both dedicated and existing staff	(*n* = 3)	Dedicated monitors and bedside staff work side by side.	USA	(38, 54, 56)
Monitoring ratio and escalation				
≤10:1 patient-to-observer	(*n* = 4)	- Ratios from 6:1 (38) to 10:1 (13, 15, 18). - Verbal redirection, then phone alert to bedside nurse.	USA	(13, 15, 18, 38)
11:1–15:1 patient-to-observer	(*n* = 11)	- Ratios of roughly 8–12:1 (12), 12:1 (11, 22, 36), 10–12:1 (33), 6–12:1 (37), and 15:1 (19, 56). - Tiered escalation; mean response times were 3.49 s (range, 0.10–28.77 s) (11).	USA	(11, 12, 19, 22, 23, 32, 33, 36, 37, 54, 56)
16:1 dashboard capacity	(*n* = 1)	- Dashboard capacity 16:1 (29); actual average 9.6:1. - Escalation to primary RN after ≥3 failed redirections.	USA	(29)
Device-capacity based, no fixed staff ratio	(*n* = 1)	- Screens show up to four patients; nurses carry devices. - Direct, immediate nurse attendance.	Australia	(31)
Automated alert direct to existing staff (no observer ratio)	(*n* = 6)	- AI-automated systems. Median staff response time to alerts was 21 s (26) - Predictive systems gave a median 28–28.5-s warning before unattended bed exit (24, 25)	USA, Malaysia, Italy, UK	(24–28, 47)

**Table 3:** Outcomes of electronic sitter interventions.

Outcome measured	Studies (n)	Key findings on the effectiveness of virtual sitting	Country	Studies
Falls	(*n* = 22)	- Significant reductions of 25%–48% (11, 23, 28, 32, 33) - Largest: 79% (27); 59% (29); adjusted OR 0.49 (56). - Weaker evidence: non-significant 25% (22); no change (36); descriptive 100% (55); bundled 54% (54); *post hoc* 83% (26)	USA, Italy, Malaysia, UK, Australia	(11, 13, 15, 18, 19, 22–29, 31–33, 35, 36, 38, 54–56)
Unassisted falls	(*n* = 5)	- 42% reduction in targeted unassisted falls (*P* = .007) (47). - 46%–54% reductions in multi-site analyses (24, 25); 862 falls averted (32).	USA	(15, 24, 25, 32, 47)
Falls with injury	(*n* = 10)	- Significant reductions of 39%–78% (22, 24, 25, 36); no serious harm in UK (28). - Non-significant: 10.5% vs. 8.5% (11); 47.4% reduction (32).	USA, UK	(11, 13, 22, 24, 25, 28, 32, 33, 36, 55)
Moderate-severity falls	(*n* = 1)	- Moderate-harm falls fell 82% (7.0–1.2 per year) (28).	UK	(28)
Unattended bed exits	(*n* = 2)	- Mobile cameras: unattended bed exits fell 89% (*P* = .018) (24). - Fixed cameras: 649% increase (*P* = .043) (24); companion +639% (25).	USA	(24, 25)
Detection accuracy	(*n* = 4)	- Depth sensor: 97.4–98.4% sensitivity; PPV 56%–59%, later >70% (24, 25). - AI optical sensor: 100% detection, 4.66% false alarms (26). - AI vision caught all five falls, no false alarms (27).	USA, Malaysia, Italy	(24–27)
Cost reduction	(*n* = 13)	- System-wide: $160,234–$3.2M (11); $2.09M/year (32). - Single-unit: $210,912/year (38); $84,000/year (54). - Payback within 12 months (19); >$800,000/year (56).	Australia, USA	(11, 13, 15, 18, 19, 23, 31–33, 38, 54–56)
Staffing and sitter hour reduction	(*n* = 16)	- Sitter hours/FTEs fell 10% (23) to 90%–91% (11, 29). - Reductions: 6.76 FTEs (38); 72% (54). - Offsets: technician shifts (33); full automation removes sitters (24–27, 47).	Italy, Malaysia, USA	(11–13, 15, 18, 23–27, 29, 33, 36, 38, 47, 54)
Technology acceptance and satisfaction	(*n* = 8)	- Satisfaction ranged 47%–97% (12), (31); 81%–85% favorable (19, 35). - 73%–100% found monitoring effective (37); usefulness rose over time (47). - Ease of use correlated with usefulness (*r* = 0.77) (12); acceptance moderate (36).	Australia, USA	(12, 19, 29, 31, 35–37, 47)
Emergency admissions, length of stay, and mortality	(*n* = 2)	- A&E admissions fell 68%; paramedic visits fell 49% (28). - Fallers stayed longer: median 12 vs. 7 days (56). - No mortality difference (12% vs. 12%) (56).	UK, USA	(28, 56)

**Table 4:** Facilitators of implementation.

Facilitating factors	Studies (n)	Key findings	Country	Studies
Leadership engagement and multidisciplinary governance	(*n* = 13)	- Multidisciplinary steering committees and unit-level nurse-manager champions coordinated rollout and accelerated adoption. - Visible executive commitment, staff involvement and IT support built buy-in and sustained investment.	USA	(11, 13, 15, 18, 22, 29, 32, 33, 35, 36, 38, 47, 54)
Structured staff training and education	(*n* = 13)	- Structured curricula and tiered superuser training supported confident adoption (15, 18, 23, 29, 38, 47). - Targeted retraining and eligibility guidance improved technician performance and appropriate use (23, 33, 55).	Australia, Italy, Malaysia, USA	(15, 18, 19, 23, 24, 26, 27, 29, 31, 33, 38, 47, 55)
Formal change-management or improvement frameworks	(*n* = 6)	- PDSA cycles iteratively refined eligibility criteria and workflow (13, 29, 54). - ADKAR (15), Concerns-Based Adoption (38) and Larrabee (37) models guided rollout, engagement and evaluation.	USA	(13, 15, 29, 37, 38, 54)
Structured eligibility algorithms and EHR/clinical-decision-support integration	(*n* = 13)	- EHR decision support and validated fall-risk criteria automated and standardized patient selection (13, 15, 32). - Pre-launch workgroups co-developed algorithms (23, 33); waitlist and nurse-driven criteria prioritized highest-need patients (11, 56).	Italy, Malaysia, USA	(11, 13, 15, 18, 19, 23, 26, 27, 32, 33, 36, 54, 56)
Privacy-preserving design and stakeholder trust-building	(*n* = 4)	- Non-recording, live-feed-only design with pause capability addressed privacy concerns and built trust (33, 35). - Purpose-built, contact-free monitoring maximized comfort and acceptance among dementia and oncology patients (27, 28).	Italy, UK, USA	(27, 28, 33, 35)
Effective escalation protocols and technician-to-bedside-staff communication	(*n* = 5)	- Tiered escalation protocols with a second responder flagged unattended high-risk patients (22, 23). - Defined technician roles and escalation education improved communication and system-wide scale-up (19, 32, 55).	USA	(19, 22, 23, 32, 55)
Data-driven feedback loops and iterative refinement	(*n* = 8)	- Fall data (38), staff feedback (15, 35) and prior-deployment outcomes (28) built leadership support. - Metrics and learning-curve data improved performance; ease of use correlated with usefulness (12, 23, 25).	UK, USA	(12, 15, 19, 23, 25, 28, 35, 38)

**Table 5:** Barriers to implementation.

Barriers and challenges	Studies (n)	Key findings	Country	Studies
Limited device or equipment supply relative to demand	(*n* = 8)	- Limited devices covered only 13% of beds (19) and 20%–25% of high-risk patients (22). - Small camera pools (5–12 units) (18, 32, 38), forced inconsistent rationing (24), waitlisting (11) and too few monitored rooms (33).	USA	(11, 18, 19, 22, 24, 32, 33, 38)
Staff resistance and preference for in-person alternatives	(*n* = 5)	- Staff preferred in-person sitters (only 12.73% rated video equal) (12) and stayed skeptical after go-live (15). - A culture shift (38) and initial eligibility uncertainty (13) were barriers; portable-monitor uptake varied (34.8% vs. 85.4%–92.9%) (31).	Australia, USA	(12, 13, 15, 31, 38)
Technology and equipment limitations	(*n* = 7)	- Software errors (11), camera-upgrade distortion (25) and single-page paging (37) undermined reliability; audio-only felt impersonal. - Off-the-shelf hardware and procurement constraints delayed rollout (31, 36); AI needed 4-week calibration (29) and better motion detection (26).	Australia, Malaysia, USA	(11, 25, 26, 29, 31, 36, 37)
Coverage gaps and under-monitoring	(*n* = 8)	- Most falls involved unmonitored patients (11, 23, 33); 90% of unassisted fallers were eligible but unmonitored (15). - Without dedicated observers, only 50.9% of alerts were attended (26, 31); intermittent and cluster monitoring proved inconsistent (35, 54).	Australia, Malaysia, USA	(11, 15, 23, 26, 31, 33, 35, 54)
Cost and resource constraints	(*n* = 6)	- Upfront implementation, staffing and hardware costs were substantial (19, 35), about $14,561 per 15-patient unit (56). - Sitter or fall savings did not fully offset costs (23, 33); ignoring equipment ROI understated true cost (18).	USA	(18, 19, 23, 33, 35, 56)
Environmental/setting-specific disruptions	(*n* = 5)	- COVID-19 limited room access (36), forced rapid rollout (18), delayed expansion (32); 36.7–37.8% of nurses deprioritized implementation (47). - Emergency department volume, acuity, layout and poor sightlines made monitoring harder than on inpatient units (22).	USA	(18, 22, 32, 36, 47)

For the full set of 25 included studies, one reviewer (AH) extracted data, and the second reviewer (GM) independently verified them against the source publications; discrepancies were resolved through discussion and correction against the original source. During data charting, any information that was missing or not reported in the included studies was recorded as “not reported” or “not available,” rather than being inferred or omitted from the charting tables.

### 2.5 Stage 5: collating, summarizing, and reporting results

In accordance with the fifth stage of the methodological framework proposed by Arksey and O'Malley (49), as enhanced by Levac et al. (50), a two-pronged approach was employed, combining a quantitative descriptive summary with a qualitative, “descriptive-analytical” synthesis, in which data were deductively coded against categories derived directly from this review's research questions. First, a quantitative descriptive summary was generated. Second, a qualitative synthesis of the extracted data was undertaken to characterize the scope of the evidence. AH led the synthesis process, and both AH and GM collaboratively reviewed the synthesized thematic categories. The process was collaborative throughout, with discrepancies resolved through iterative discussion between the two reviewers. The synthesis describes the characteristics of the included studies, the taxonomy of electronic sitter technologies and interventions, the outcomes of electronic sitter interventions, and the facilitators of and barriers to their implementation, giving an overarching perspective on the literature landscape in line with the aim of this study.

## 3 Results

### 3.1 Study characteristics

Twenty-five studies met the inclusion criteria. Most were conducted in the United States of America (USA) (*n* = 21, 84%), with one each from Malaysia (26), Italy (27), Australia (31), and the UK (28). Settings were most often medical-surgical units (*n* = 6) (12, 13, 33, 37, 47, 54), rehabilitation or brain-injury units (*n* = 3) (19, 29, 35), multiple units in one institution (*n* = 3) (18, 23, 38), or multiple hospitals (*n* = 5) (15, 24, 25, 32, 36). Other settings included an emergency department (22) and a dementia care unit (28). Samples ranged from 30 patients to over 174,000 visits. Nine studies included staff (12, 28, 29, 31, 35–37, 47, 55). Quality-improvement or evidence-based practice projects predominated (*n* = 9) (13, 15, 18, 22, 29, 35, 38, 54, 55), followed by cohort (*n* = 6) (11, 19, 27, 28, 31, 56) and quasi-experimental (*n* = 4) (32, 33, 36, 47) designs; single studies used a multi-site pretest-posttest design (24), a technical case study (25), a descriptive pre-post evaluation (23), a single-arm pilot (26), a staff survey (12), and mixed methods (37). Only two had concurrent comparison groups (27, 32) (Table 1).

### 3.2 Taxonomy of electronic sitter technologies and interventions

Electronic sitter technologies and interventions were classified across seven dimensions: vendor platforms, sensing modality, observation model (continuous vs. event-triggered), fall detection (human vs. AI-assisted), monitoring location (centralized vs. unit-based), observer type (dedicated vs. bedside staff), and monitoring ratio and escalation (Table 2). By technology or vendor platform, AvaSure systems (*n* = 3) (11, 22, 23) and the Ocuvera automated video monitoring system (*n* = 3) (24, 25, 47) were most frequent. Six further platforms were each evaluated once: the SMART AI Patient Sitter (26), Verso Vision (27), Oxehealth (28), Artisight-enabled virtual patient observation with a Virtual Nurse (29), Epic Monitor (13), and repurposed Uniden baby monitors (31). Thirteen studies did not name the platform for CVM/RVM (12, 15, 18, 19, 32, 33, 35–38, 54–56).

For sensing modality, standard camera or video technology predominated (*n* = 20) (11–13, 15, 18, 19, 22, 23, 27, 29, 31–33, 35–38, 54–56). Depth sensors were used in three studies of one platform (24, 25, 47), whereas multimodal optical–thermal imaging (26) and non-displaying infrared computer vision (28) each appeared once. For observation delivery, continuous human observation of a live feed was dominant (*n* = 16) (11–13, 15, 18, 19, 22, 23, 32, 33, 36–38, 54–56). Event-triggered review, in which automated alerts prompted human review, occurred in eight studies (24–29, 31, 47), and one study used intermittent, non-automated review by existing unit staff (35). For fall detection, most studies relied solely on human visual judgment (*n* = 17) (11–13, 15, 18, 19, 22, 23, 32, 33, 35–38, 54–56). Seven studies used AI-assisted detection (24–29, 47), and one used rule-based motion- and sound-triggered alerts without algorithms (31). Danial et al. (26) used a residual network (ResNet)-based convolutional neural network to detect bed exits and falls and a You Only Look Once (YOLO)-based model to recognize staff (Table 2).

By location, monitoring was most often centralized in a dedicated room or off-site center serving multiple units (*n* = 16) (11–13, 15, 18, 19, 22, 23, 26, 29, 32, 33, 36–38, 56). Unit- or ward-based monitoring (*n* = 4) (28, 35, 47, 55) and decentralized models, with alerts sent to staff mobile devices or devices carried on rounds (*n* = 4) (24, 25, 27, 31), were less common. For staffing, a dedicated observer role, such as a video monitor technician or telesitter, was created in 14 studies (11–13, 15, 18, 19, 22, 23, 29, 32, 33, 36, 37, 55), whereas existing bedside staff responded in eight (24–28, 31, 35, 47), and three combined both (38, 54, 56). Patient-to-observer ratios ranged from 6:1 to 15:1, with 11:1 to 15:1 reported most frequently (*n* = 11) (11, 12, 19, 22, 23, 32, 33, 36, 37, 54, 56); one study reported a dashboard capacity of 16:1 (29), and four reported ratios of 10:1 or lower (13, 15, 18, 38). In six studies, automated alerts went directly to existing staff without a human observer (24–28, 47). Escalation was typically tiered, progressing from verbal redirection via two-way audio to notifying the bedside nurse and, if unresolved, triggering an alarm or emergency response (11, 12, 19, 22, 23, 32, 33, 36, 37, 54, 56); the mean technician response time was 3.49 s (range, 0.10–28.77 s) (11). Among automated systems (24–28, 47), median staff response time to alerts was 21 s (26), and predictive systems gave a median warning of 28–28.5 s before an unattended bed exit (24, 25).

### 3.3 Outcomes of electronic sitter interventions

Outcomes were mapped across 10 categories: fall incidence, unassisted falls, falls with injury, moderate-severity falls, unattended bed exits, detection accuracy, cost reduction, staffing and sitter hour reduction, technology acceptance and satisfaction, and emergency admissions, length of stay, and mortality (Table 3). Most studies reported more than one outcome; however, outcome definitions, denominators, observation periods, and analytic approaches varied considerably, and most evaluations used single-site, uncontrolled pre-post or quality-improvement designs. Consistent with a scoping review approach, findings are therefore synthesized descriptively to characterize the extent and nature of the evidence, rather than pooled to estimate effectiveness.

#### 3.3.1 Fall incidence

Falls were the most frequently reported outcome (*n* = 22) (11, 13, 15, 18, 19, 22–29, 31–33, 35, 36, 38, 54–56), and most studies from the USA, Italy, Malaysia, the UK, and Australia reported fewer falls after implementation. Statistically significant reductions were observed across human-staffed, AI-augmented, and hybrid models, ranging from 25.1% (*P* = .02) (11) to 79% [incidence rate ratio (IRR): 0.21, 95% confidence interval (CI): 0.12–0.38, *P* < .0001] (27), with most between 28.5% and 48% (23, 28, 32, 33); post-implementation admission was also associated with lower adjusted odds of falling [odds ratio (OR) 0.49, *P* < .001] (56). However, the strength of the evidence was uneven. Reductions of 54%–100% were supported only by descriptive comparison, statistical process control, or *post hoc* power analysis (26, 29, 54, 55), and the effect of video monitoring could not be isolated within multicomponent bundles (22, 54). Two evaluations found a non-significant 25% reduction (*P* = .18) (22) or essentially no change in fall rates (36). Benefits also appeared concentrated in high-risk patients (*P* < .001, compared with *P* = .301 in low- to moderate-risk patients) (32) and on particular wards (31), and residual falls clustered among unmonitored patients (11, 15, 23, 26, 31, 33, 35, 54); in one study, 68.3% of falls involved patients who did not meet 1:1 sitter criteria (11). Effectiveness therefore appeared bounded by device coverage and responder capacity as much as by the technology itself.

#### 3.3.2 Unassisted falls

Unassisted falls were reported in five USA studies (15, 24, 25, 32, 47). Only one study, which targeted bed-origin falls with an automated system, reported a statistically significant reduction of 42% (1.71–1.0 per 1,000 patient-days, *P* = .007) (47), whereas a larger multi-site evaluation found a non-significant 54% reduction (*P* = .214) (24) and its companion pooled analysis reported a consistent decrease of approximately 46%–54% (25). The remaining evidence was indirect: 90% of patients with unassisted falls met virtual-observation eligibility criteria in one study (15), and an estimate of 862 averted falls in another was extrapolated rather than directly measured (32). Inconsistent terminology, with some studies reporting “unwitnessed” rather than “unassisted” falls, further limited comparability.

#### 3.3.3 Falls with injury

Ten studies from the USA and the UK reported falls with injury (11, 13, 22, 24, 25, 28, 32, 33, 36, 55), mostly using National Database of Nursing Quality Indicators (NDNQI) or Agency for Healthcare Research and Quality (AHRQ) definitions. Statistically significant reductions were confined to a minority, ranging from 39.15% (*P* = .006) (36) to 66% (*P* < .05) (22), alongside a shared 78% reduction (*P* = .025) across automated-system deployments (24, 25). Two studies reported zero injurious falls among video-monitored patients (13, 33), whereas other changes did not reach significance, including 10.5% versus 8.5% (*P* = .39) (11) and a 47.4% reduction (*P* = .432) (32). Because injurious falls were rare events in mostly small, single-site samples, studies were generally underpowered, and severity breakdowns were inconsistently reported, with one UK study using a facility-specific harm scale that recorded no serious harm (28). The evidence therefore suggests a favorable direction of effect on fall-related harm, but with limited certainty.

#### 3.3.4 Moderate-severity falls

Only one UK study distinguished moderate-severity falls, using a facility-specific rather than an NDNQI-based harm scale (28). It reported an 82% reduction in moderate-harm falls, from an annualized rate of 7.0–1.2 (28). Although this reduction was substantial, reliance on a single study and a non-standard classification means that the effect of electronic sitters on fall severity remains largely unexamined.

#### 3.3.5 Unattended bed exits

Unattended bed exits were examined in two USA analyses of the same automated depth-sensor platform (24, 25), and outcomes depended on deployment configuration. Exits fell by 89% at sites using mobile cameras that allowed staff to intervene (*P* = .018) but increased paradoxically by 649% at fixed-camera sites (*P* = .043) (24), with the companion analysis reporting consistent figures (−89% and +639%) (25). The authors attributed the increase to expanded surveillance capturing low-risk patients safely leaving bed (24), indicating that process measures captured by monitoring systems may reflect surveillance intensity rather than changes in patient risk.

#### 3.3.6 Detection accuracy

Detection accuracy was reported only for AI-augmented platforms (*n* = 4) evaluated in the USA, Malaysia, and Italy (24–27). One depth-sensor system achieved 97.4–98.4% sensitivity, with positive predictive value improving from 56.4%–59.4% in early deployments to over 70% later and median alert lead times of 28–28.5 s (24, 25), indicating that algorithmic performance matured with local use and refinement. An AI optical-sensor system reported 100% detection, a 4.66% false-alarm rate, 95.34% accuracy, and a 21-s median response time (26), and an AI computer-vision system flagged all five falls without false-positive alerts, although this was reported qualitatively (27). Because these findings derive from few evaluations using heterogeneous metrics, the generalizability of reported accuracy to other settings remains uncertain.

#### 3.3.7 Cost reduction

Cost outcomes were reported in 13 studies from the USA and Australia (11, 13, 15, 18, 19, 23, 31–33, 38, 54–56) and generally indicated that savings from reduced 1:1 sitter use exceeded implementation costs. Savings scaled with the level of deployment. System-level projections were the largest, ranging from more than $800,000 annually (56) and nearly $1 million after the first year (15) to $2.09 million annually (32) and up to $3.2 million when fall-related costs were included (11). Single-unit programs reported more modest savings, typically between approximately $84,000 and $226,000 (18, 19, 38, 54), with payback within 12 months to two years (18, 19), and as little as $12,097 in one program (13). Net benefit was not universal, as savings were partly or fully offset by the cost of new monitoring staff in some programs (23, 33), and one study reported equipment costs without estimating savings (31). Cost estimates relied heavily on projections and differing assumptions, and inconsistently included equipment, staffing, and fall-related costs, which limits comparability across studies.

#### 3.3.8 Staffing and sitter hour reduction

Staffing outcomes were reported in 16 studies from the USA, Italy, and Malaysia (11–13, 15, 18, 23–27, 29, 33, 36, 38, 47, 54). Programs using dedicated observers reported reductions in 1:1 sitter hours or full-time equivalents (FTEs) ranging from 10% (23) to 90%–91% (11, 29), including 4,879.5 net sitter hours saved (18), a 6.76 FTE reduction (38), and 72% less sitter use (54), while one study estimated that 42.7% of technicians could be freed from 1:1 assignments (15). These gains were, however, partly offset by the new observer workforce: sitter-shift savings were offset by new technician shifts in one study (33), a 12:1 central program required 51.6 FTEs (36), and nurses were still pulled from staffing 65.45% of the time in another (12). Fully automated systems that routed alerts to existing nurses avoided dedicated sitter staffing altogether but did not quantify the additional workload placed on bedside staff (24–27, 47).

#### 3.3.9 Technology acceptance and satisfaction

Eight studies from the USA and Australia assessed staff perceptions (12, 19, 29, 31, 35–37, 47), mostly through researcher-developed surveys, with few using validated technology-acceptance instruments (12, 36, 47). Reported perceived value or satisfaction varied widely, from 47.27% (12) to 97% (31), although most studies reported favorable views, with 81% of staff reacting favorably (19), 85% rating monitoring as valuable (35), and 73%–100% believing video monitoring was effective (37). Acceptance was moderately positive using the Remote Visual Monitoring Acceptance Tool (RVMAT), with a mean of 3.38/5 (36) and increased with exposure, as perceived usefulness rose significantly across survey waves [*t*(10) = 4.74, *P* < .05] (47); perceived ease of use was also strongly correlated with usefulness (*r* = 0.77) (12). Acceptance was not unequivocal, however, as staff in one study preferred in-person sitters over video monitoring (*P* = .0003) (12). Patient and family perspectives were rarely captured, with only one study reporting patient-experience outcomes (29).

#### 3.3.10 Emergency admissions, length of stay, and mortality

Two studies from the UK and the USA reported healthcare utilization and clinical outcomes beyond falls (28, 56). In a UK dementia inpatient setting, accident and emergency admissions fell significantly by 68% (*P* < .02), whereas the 49% reduction in paramedic visits was not statistically significant (*P* < .2) (28). In a USA study, patients who fell had a longer length of stay than those who did not (median 12 vs. 7 days, *P* < .001) but similar mortality (12% vs. 12%, *P* > .9) (56), indirectly linking fall prevention to hospital utilization. No study reported hospital readmission.

### 3.4 Facilitators of electronic sitter implementation

Facilitators spanned seven overlapping domains, and most studies reported more than one (Table 4). Leadership and multidisciplinary governance were the most frequently cited (*n* = 13), operating through cross-departmental steering committees, executive sponsorship, unit champions, and explicit leadership communication and commitment (11, 13, 15, 18, 22, 29, 32, 33, 35, 36, 38, 47, 54). Equally common was structured education (*n* = 13), which ranged from brief orientation to 8-h, 3-day, or multi-week programs, often with tiered superuser models; alarm-fatigue training, data-informed retraining, and written documentation supported performance, whereas low training burden was itself a facilitator for portable and computer-vision systems (15, 18, 19, 23, 24, 26, 27, 29, 31, 33, 38, 47, 55). Implementation was further anchored in a named improvement framework in six studies, including ADKAR, the Concerns-Based Adoption Model, the Larrabee Model, and a Kaizen event with external coaching (13, 15, 29, 37, 38, 54), and PDSA cycles were most common (13, 29, 54).

At the operational level, 13 studies cited structured eligibility algorithms or EHR decision support, including Morse-triggered alerts, eligibility reports, decision trees, embedded fall-risk instruments, device wait-lists, and explicit initiation and discontinuation criteria, typically co-developed by interdisciplinary groups and refined iteratively (11, 13, 15, 18, 19, 23, 26, 27, 32, 33, 36, 54, 56). Acceptance was supported by privacy-preserving design in four studies, including non-recording cameras, pause/block safeguards, and contact-free sensing suited to people with dementia and well accepted by medical and oncology patients (27, 28, 33, 35). To ensure timely bedside response, five studies credited clear escalation and communication protocols, including designated second responders, fallback alarms or in-person observers when devices were fully used, defined technician roles, and escalation training (19, 22, 23, 32, 55). Finally, eight studies fed data back into programs, using fall data to address staff concerns, staff and family feedback to sustain investment and guide vendor selection, performance metrics to refine AI systems, and prior deployment outcomes (755 nights without adverse events) to support expansion (12, 15, 19, 23, 25, 28, 35, 38).

### 3.5 Barriers to electronic sitter implementation

Barriers spanned six overlapping domains, and most studies reported more than one (Table 5). The most consistently quantified was insufficient device supply (*n* = 8), necessitating waitlisting and reassignment and sometimes diverting fixed cameras to lower-risk patients (11, 18, 19, 22, 24, 32, 33, 38). Only 2 of 36 devices served the emergency department, covering 20%–25% of high-risk patients (22); 15 of 115 beds (13%) could be monitored at once (19); and only 10 (18) or 12 (32) cameras supported hospital- or system-wide rollouts. Beyond supply, five studies reported staff resistance or preference for in-person sitters, including the need for a “culture shift,” persistent skepticism, and uncertainty about eligibility (12, 13, 15, 31, 38). Only 12.73% of staff considered video monitoring as effective as a sitter (12). Portable-monitor uptake was 34.8% on one ward versus 85.4%–92.9% on two others, with lower alert response on the low-uptake ward (31). Coverage gaps and under-monitoring were reported in eight studies, with falls clustering among eligible patients who were awaiting a device, not yet connected, or outside camera view (11, 15, 23, 26, 31, 33, 35, 54). Technology limitations further constrained use in seven studies, including impersonal audio, camera image distortion, single-page paging, consumer-grade hardware constraints, wireless and procurement delays, four-week AI calibration, and difficulty detecting rapid movements (11, 25, 26, 29, 31, 36, 37), and software errors caused 33 of 447 discontinuations (7.4%) (11).

Cost was a further barrier in six studies, including upfront implementation and staffing costs, savings that did not fully offset new staffing costs, and analyses omitting equipment costs (18, 19, 23, 33, 35, 56), and hardware costs were approximately $14,561 (2020 USD) per 15-patient unit (56).

Five studies reported disruption from COVID-19, which limited room access, forced accelerated rollouts, and halted data collection, or from emergency department layout and volume (18, 22, 32, 36, 47). During pandemic surges, 36.7–37.8% of nurses reported that implementation was not a priority (47).

## 4 Discussion

Across 25 studies, electronic sitters showed potential for scalable reductions in falls and costs, but the benefit was conditional. Human-staffed continuous video monitoring predominated (11–13, 15, 18, 19, 22, 23, 32, 33, 36–38, 54–56), with AI-supported systems in a minority (24–29, 47), at ratios of 6:1–15:1 (11, 12, 19, 22, 23, 32, 33, 36, 37, 54, 56) and a maximum capacity of 16:1 in one system (29). AI-augmented systems detected bed exits with 97.4–98.4% sensitivity and 28–28.5 s lead times (24, 25), consistent with the 93% deep-learning accuracy reported by Taramasco et al. (5), and Hruby et al. (4) confirmed that unobtrusive camera- and depth-sensor systems monitor falls without disruption. Electronic sitter programs differed less in sensors than in how work was organized around them: who watches, how alerts escalate, who responds, and how the program is integrated into the existing staffing and fall-prevention workflows (18). A sociotechnical systems approach to patient journey and safety highlights the need for multiple perspectives, with technology, people, tasks, and context designed jointly (57). In health technology implementation, realist Context–Mechanism–Outcome (CMO) thinking explains why and how outcomes vary across settings. Context encompasses the conditions in which a technology is introduced, including organizational arrangements, professional norms, available resources, workflow characteristics, and the wider institutional environment (58–61). Technologies provide resources and opportunities that, in particular contexts, shape users’ reasoning and responses. These resource–reasoning interactions, rather than technical features alone, constitute mechanisms (59). These interactions help explain different implementation trajectories, including adoption, adaptation, nonadoption, abandonment, and workarounds, with potential implications for patient safety and clinical efficiency (58, 61, 62). Technology-related working conditions may also affect staff well-being, and poor usability of digital health tools is associated with burnout (63).

Electronic sitters reached a broader range of settings and populations at risk of falls, including emergency departments (22), dementia wards (28), and rehabilitation units (19, 29, 35). Fall-risk instruments are commonly used to assess patients, including the Morse Fall Scale (13, 56), the Johns Hopkins Fall Risk Assessment Tool (32, 55), and the Hendrich II (33). Additional eligibility drivers included confusion, agitation, and COVID-19 isolation status (11, 15, 18, 35, 47). In this review, significant fall reductions ranged from 25.1% (11) to 79% (27), yet the largest effects rested on weak designs: an 83.33% reduction from *post hoc* power analysis (26) and six-week elimination that was descriptive (55). Only four studies showed significant injury reductions (22, 24, 25, 36).

Evidence is promising in falls prevention and reduction but inconsistent, a pattern also seen in mental health surveillance and wearable sensing, where Griffiths et al. (64) and Kristoffersson and Linden (65) found weak evidence. Video review can nonetheless support secondary prevention, as Bayen et al. (34) showed in dementia care. Three mechanisms may explain the inconsistency. Monitoring alters vigilance and documentation (the Hawthorne effect) (66), as the paradoxical rise in recorded unattended bed exits suggests (24, 25). Effects depend on context (67), with device coverage and responder capacity determining whether alerts become interventions (11, 15, 23, 26, 31, 33, 35, 54). And because sitters are complex interventions (68), bundled components (22, 54) cannot be separated in uncontrolled pre-post designs. Given annual fall costs of $19.8 billion in the USA (69), even conditional benefits warrant rigorous evaluation. Economic gains were large but largely projected: sitter-hour reductions reached 90%–91% (11, 29) and savings ranged from $88,000 over six weeks (55) to $3.2 million over 20 months (11), consistent with remote-monitoring evidence (8). Acceptance was uneven: 73%–100% of staff believed monitoring prevented falls (37), and 97% rated a portable system positively (31), yet only 12.73% considered video as effective as a bedside sitter (12). Acceptance therefore cannot be assumed.

Hesitancy has several plausible explanations. Technology acceptance models link adoption to usefulness, ease of use, social influence, and facilitating conditions (70, 71). In this review, leadership champions supplied social influence (11, 13, 15, 18, 22, 29, 32, 33, 35, 36, 38, 47, 54), whereas device scarcity (11, 18, 19, 22, 24, 32, 33, 38) and software or hardware faults (11, 25, 26, 29, 31, 36, 37) eroded facilitating conditions. Because such models treat adoption as individual judgment, a preference for in-person sitters (12, 13, 15, 31, 38) may also reflect relational presence that remote observation replicates only partially (72) and role displacement (73). Camera surveillance strains dignity and autonomy, especially in dementia (74); non-recording design built trust (27, 28, 33, 35), and Mateus et al. (30) argue that AI surveillance requires explicit ethical frameworks covering consent, data security, and autonomy.

Uptake depended on implementation context. Software errors caused 7.4% of discontinuations in one program (11), AI calibration took four weeks of local adaptation (29), and camera-generation hardware transitions introduced image distortion (25). Diffusion of innovations theory predicts adoption from trialability and observability (75); phased rollouts alongside existing sitters supported staff confidence (35, 47), whereas scarce devices limited observability. Furthermore, the diffusion or non-diffusion of innovations can be understood in terms of three interacting domains: perceived innovation attributes; adopter characteristics and interpersonal influences (particularly those of opinion leaders); and the wider social and political context, including issue salience, stakeholder framing, and introduction timing (75). The nonadoption, abandonment, scale-up, spread, and sustainability (NASSS) framework attributes abandonment to misalignment across domains (58), as pandemic disruption showed (18, 32, 36, 47), and normalization process theory requires reflexive monitoring (76), which few programs reported (12, 15, 19, 23, 25, 28, 35, 38). Frameworks such as PDSA (13, 29, 54), ADKAR (15), and the Concerns-Based Adoption Model (38) operationalize these constructs (75, 77), and Lewis et al. (48) identified nursing leadership and governance as foundational. As Piotrowicz et al. (40) noted, technology is a delivery mechanism, not the intervention. Practical strategies follow: sharing local outcome data with staff, securing device supply, and providing tiered training addressing alarm fatigue (15, 18, 19, 23, 24, 26, 27, 29, 31, 33, 38, 47, 55) and automation bias (78, 79).

To safeguard relational care, electronic sitting should supplement, not replace, bedside nursing, selectively substituting one-to-one observation only when clinically appropriate. Bedside nurses should help develop eligibility criteria, reassessment procedures, and escalation pathways (80). To foster patient and family confidence, privacy governance must be explicit, providing clear information on monitoring and safeguards during personal care (80). These governance arrangements should be co-developed with patients and families to address concerns regarding privacy, dignity, and therapeutic relationships (64). Implementation barriers could be systematically assessed using the updated Consolidated Framework for Implementation Research (CFIR) (77) and linked to potential strategies via structured, context-sensitive selection methods such as CFIR–ERIC (Expert Recommendations for Implementing Changes Compilation) matching (81). Prospectively specifying chosen strategies and relevant clinical and implementation outcomes would facilitate their evaluation in future hybrid effectiveness–implementation studies (82).

## 5 Limitations and recommendations

Several limitations warrant consideration when interpreting the findings of this review. The search was confined to English-language publications, so relevant evidence in other languages may be missing. Most included studies were conducted in the United States, limiting transferability to other health-system and resource contexts, particularly in low- and middle-income settings. Consistent with its evidence-mapping purpose, this review did not exclude studies based on MMAT appraisal; the findings should be read as a map of the available evidence rather than definitive estimates. Heterogeneity in intervention designs, eligibility criteria, and outcome definitions limits comparability and prevents quantitative pooling. Outcomes were inconsistently defined, including total falls, unassisted falls, fall-related injuries, unattended bed exits, and detection accuracy, preventing firm conclusions about effectiveness.

Most studies used uncontrolled pre-post designs, and only two (27, 32) included a concurrent comparison group; improvements cannot be separated from regression to the mean, secular trends, surveillance effects, or co-interventions—several studies combined video monitoring with a wider fall-prevention bundle, such as mobility assessment, medication review, and hourly rounding in Spano-Szekely et al. (54)—making the independent contribution of monitoring difficult to isolate. Vendor involvement was notable, with a financial relationship between authors or sponsors and the technology vendor in at least five studies: Allen et al. (47), Jones et al. (24) and the companion Kramer et al. (25), Danial et al. (26), Tyransky et al. (29), and Votruba et al. (23), which may affect interpretation of their findings. This review also gives limited attention to patient and family perspectives, informed consent, equity of access, and alarm burden on staff.

Future research should use randomized controlled or stepped-wedge designs with adequately powered samples and standardized definitions of falls, fall-related injuries, and sitter utilization to enable meta-analysis. Economic evaluations, hybrid effectiveness–implementation studies, and research in resource-constrained settings are also needed. Implementers should embed ethical frameworks addressing consent, privacy, and data governance, and report fidelity, coverage gaps, and adverse events transparently. This review did not systematically examine commercial influence on the evidence. Funding sources, vendor participation, conflicts of interest, equipment provision, and author affiliations were not consistently assessed across included studies. Future studies should report and appraise these sources transparently.

## 6 Conclusion

This scoping review synthesized 25 primary studies examining electronic sitters—including human-staffed continuous video monitoring, AI-augmented virtual observation, and consumer-grade portable devices—to prevent falls and fall-related injuries among hospitalized patients. The evidence indicates that these technologies substantially extend observation capacity, enabling a single trained technician to monitor between six and fifteen high-risk patients concurrently in typical use, clustering around ten to twelve, with one system's maximum technical capacity reported at sixteen patients. Well-implemented programs reported statistically significant reductions in fall rates ranging from 25.1% to 79%, reductions in sitter hours of 90%–91% in some studies, and institutional savings of up to US$3.2 million over 20 months when avoided fall-related costs were included. Nevertheless, these findings warrant cautious interpretation, as estimates were heterogeneous, net savings were not universal, injury-related outcomes were inconsistently evaluated (only four of ten studies reported significant reductions), and predominantly uncontrolled pre-post designs, with only two studies using concurrent comparison groups, preclude robust causal inference.

A consistent finding across the included studies is that the monitoring technology, on its own, does not constitute the intervention. Observed benefits were contingent upon the organizational and clinical infrastructure surrounding deployment—multidisciplinary governance, structured staff training, eligibility algorithms embedded within the electronic health record, disciplined escalation protocols, and privacy-preserving design capable of securing the trust of patients, families, and nursing staff. Where device supply, staff engagement, or monitoring coverage was inadequate, surveillance documented risk without averting it. Electronic sitters are therefore best conceptualized as complex sociotechnical interventions, requiring implementation rigor comparable to other clinical programs and evaluation of greater methodological quality. For health systems confronting workforce shortages and the escalating clinical and economic burden of inpatient falls, adoption decisions about these technologies should depend on local needs, infrastructure, patient population, ethical safeguards, and prospective evaluation, rather than on an assumption that electronic sitters are broadly effective or suitable across all settings.

## References

[1] LeLaurin JH Shorr RI. Preventing falls in hospitalized patients: state of the science. Clin Geriatr Med. (2019) 35(2):273–83. 10.1016/j.cger.2019.01.007 30929888 PMC6446937

[2] Li S Surineni K. Falls in hospitalized patients and preventive strategies: a narrative review. Am J Geriatr Psychiatry Open Sci Educ Pract. (2025) 5:1–9. 10.1016/j.osep.2024.10.004

[3] WHO. Falls (2021). Available online at: https://www.who.int/news-room/fact-sheets/detail/falls (Accessed October 12, 2026).

[4] Hruby D Hruba E Cerny M. Research of fall detection and fall prevention technologies: a review. Sensors. (2026) 26(4):1–24. 10.3390/s26041192 PMC12943967 41755133

[5] Taramasco C Lazo Y Rodenas T Fuentes P Martínez F Demongeot J. System design for emergency alert triggered by falls using convolutional neural networks. J Med Syst. (2020) 44(50):1–13. 10.1007/s10916-019-1484-1 31907688

[6] Oliver D Healey F Haines TP. Preventing falls and fall-related injuries in hospitals. Clin Geriatr Med. (2010) 26(4):645–92. 10.1016/j.cger.2010.06.005 20934615

[7] Visvanathan R Ranasinghe DC Wilson A Lange K Dollard J Boyle E. Effectiveness of an ambient intelligent geriatric management system (AmbIGeM) to prevent falls in older people in hospitals: protocol for the AmbIGeM stepped wedge pragmatic trial. Inj Prev. (2019) 25(3):157–65. 10.1136/injuryprev-2017-042507 28823995

[8] Tan SY Sumner J Wang Y Wenjun Yip A. A systematic review of the impacts of remote patient monitoring (RPM) interventions on safety, adherence, quality-of-life and cost-related outcomes. npj Digit Med. (2024) 7(1):1–16. 10.1038/s41746-024-01182-w 39025937 PMC11258279

[9] Hima Bindu K Haranath SP Mahajan RP Subba Reddy K Khandelwal R Varahala S. Continuous patient monitoring in healthcare: a comprehensive review of opportunities, challenges, and future directions. Telehealth Med Today. (2025) 10(1):1–8. 10.30953/thmt.v10.562

[10] Greeley AM Tanner EP Mak S Begashaw MM Miake-Lye IM Shekelle PG. Sitters as a patient safety strategy to reduce hospital falls: a systematic review. Ann Intern Med. (2020) 172(5):317–24. 10.7326/m19-2628 32016286

[11] Kalafus L Hrdlicka H Lombardi J Proctor S Napolitano J Morrill N. Implementation of a continuous video monitoring program to decrease inpatient falls in a long-term acute care hospital setting: a prospective observational cohort study. Am J Nurs. (2025) 125(12):20–7. 10.1097/AJN.0000000000000197 41261482 PMC12643557

[12] Hebb A Kistler M George E Zamboni B. Satisfaction and technology acceptance of staff regarding use of continuous video monitoring in comparison with sitters. J Nurs Adm. (2021) 51(2):60–2. 10.1097/NNA.0000000000000970 33449593

[13] Charles MZ. Implementing a virtual sitter program to reduce patient falls and cost (Dissertation/thesis). University of Alabama in Huntsville, Huntsville, AL, United States (2023). Available online at: https://louis.uah.edu/uah-dnp/61/ (Accessed June 3, 2026).

[14] Li A Goldberg SA Kennedy M Hayden E Loughlin KM Ouchi K. Virtual observation units: a novel disposition for older adults with falls from the emergency department. JACEP Open. (2025) 6(5):1–6. 10.1016/j.acepjo.2025.100230 PMC12396014 40894190

[15] Sacks N Wong Q. Leveraging virtual observation technology to transform patient care and reduce falls. Nurs Manage. (2025) 56(9):37–42. 10.1097/nmg.0000000000000306 41172009

[16] Chaytee J Dinh A D’Anglejan E Bouchand F Jaffal K Duran C. Digital health for remote home monitoring of patients with COVID-19 requiring oxygen: a cohort study and literature review. Front Med (Lausanne). (2023) 10:1–9. 10.3389/fmed.2023.1255798 PMC10864516 38356737

[17] Lee S Lim JJ Kourounis G Cheong J Courtney M. Remote patient monitoring following same-day discharge bariatric surgery: a systematic review and meta-analysis. Obes Surg. (2025) 35(4):1357–76. 10.1007/s11695-025-07756-1 40000568

[18] Hogan Quigley B Renz SM Bradway C. Fall prevention and injury reduction utilizing continuous video monitoring: a quality improvement initiative. J Nurs Care Qual. (2022) 37(2):123–9. 10.1097/NCQ.0000000000000582 34231506

[19] Cournan M Fusco-Gessick B Wright L. Improving patient safety through video monitoring. Rehabil Nurs. (2018) 43(2):111–5. 10.1002/rnj.308 29499009

[20] Sang S Barnes C McDonnell TM Phinney D Lytle KS Padilla BI. Staffing, workflow, and operational models in virtual nursing and remote safety observation: a scoping review. Nurs Outlook. (2026) 74(4):102804. 10.1016/j.outlook.2026.102804 42190513

[21] Sharma A Rastogi D Pandey MS. A detailed review on potential role of Internet of Medical Things (IoMT) in healthcare. Int J Syst Assur Eng Manag. (2026):1–16. 10.1007/s13198-026-03383-w

[22] Cook N Komansky B Urton M. Do no harm: a multifactorial approach to preventing emergency department falls-a quality improvement project. J Emerg Nurs. (2020) 46(5):666–74. 10.1016/j.jen.2020.03.007 32507724

[23] Votruba L Graham B Wisinski J Syed A. Video monitoring to reduce falls and cpatient companion costs for adult inpatients. Nurs Econ. (2016) 34(4):185–9. Available online at: https://pubmed.ncbi.nlm.nih.gov/29975024/ 29975024

[24] Jones KJ Haynatzki G Sabalka L. Evaluation of automated video monitoring to decrease the risk of unattended bed exits in small rural hospitals. J Patient Saf. (2021) 17(8):E716–26. 10.1097/PTS.0000000000000789 33009181 PMC8612902

[25] Kramer JB Sabalka L Rush B Jones K Nolte T. Automated depth video monitoring for fall reduction: a case study. In: Proceedings of the IEEE/CVF Conference on Computer Vision and Pattern Recognition (CVPR) Workshops (2020). p. 1188–96. 10.1109/CVPRW50498.2020.00155

[26] Danial M Chow CT Lim MH Ayop NA Looi I Ch'ng ASH. AI-based patient monitoring for fall prevention in stroke patients: a pilot study at a Malaysian acute stroke unit. J Neuroeng Rehabil. (2025) 22(1):216. 10.1186/s12984-025-01706-9 41121354 PMC12542081

[27] Gervasi C Perego E Galli F Torri V Castoldi M Bombardieri E. Prevention of falls in hospitalized patients—evaluation of the effectiveness of a monitoring system (Verso Vision) developed with artificial intelligence. Front Digit Health. (2025) 7:1–7. 10.3389/fdgth.2025.1548209 PMC12040876 40309322

[28] Wright K Singh S. Reducing falls in dementia inpatients using vision-based technology. J Patient Saf. (2022) 18(3):177–81. 10.1097/PTS.0000000000000882 34347740 PMC9359773

[29] Tyransky CE Paulus K Langmead E Miller DM Smith CR Hughes F. Integration of virtual technology and artificial intelligence improves satisfaction, patient safety, and nursing workforce efficiency. J Nurs Care Qual. (2025) 40(3):193–201. 10.1097/NCQ.0000000000000842 39999215

[30] Mateus M Alho I Neves AL Lopes H Correia M. “First, do no harm” in the digital era: examining the practicality of the European Health Data Space proposal and ethical implications of artificial intelligence: a systematic literature review. BMC Med Ethics. (2026) 27(1):1–26. 10.1186/s12910-025-01372-5 PMC12927267 41519775

[31] Woltsche R Mullan L Wynter K Rasmussen B. Preventing patient falls overnight using video monitoring: a clinical evaluation. Int J Environ Res Public Health. (2022) 19(21):1–12. 10.3390/ijerph192113735 PMC9657748 36360616

[32] Zimbro KS Bridges C Bunn S Wilmoth DD Beck M Smith CV. Remote patient monitoring improves patient falls and reduces harm. J Nurs Care Qual. (2024) 39(3):212–9. 10.1097/NCQ.0000000000000749 37782901

[33] Sand-Jecklin K Johnson JR Tylka S. Protecting patient safety: can video monitoring prevent falls in high-risk patient populations? J Nurs Care Qual. (2016) 31(2):131–8. 10.1097/NCQ.0000000000000163 26513398

[34] Bayen E Jacquemot J Netscher G Agrawal P Tabb Noyce L Bayen A. Reduction in fall rate in dementia managed care through video incident review: pilot study. J Med Internet Res. (2017) 19(10):e339. 10.2196/jmir.8095 29042342 PMC5663952

[35] Baez M Karl C. Preventing falls with remote video monitoring. Nursing (Brux). (2022) 52(7):47–9. 10.1097/01.NURSE.0000832384.85335.43 35752910

[36] Ergai A Spiva L Thurman S Hatfield M McCollum M Holmes M. The effectiveness of remote video monitoring on fall prevention and nurses’ acceptance. J Nurs Care Qual. (2024) 39(1):24–30. 10.1097/NCQ.0000000000000716 37094580

[37] Sand-Jecklin K Johnson J Tringhese A Daniels C White F. Video monitoring for fall prevention and patient safety: process evaluation and improvement. J Nurs Care Qual. (2019) 34(2):145–50. 10.1097/NCQ.0000000000000355 30198947

[38] Purvis S Kaun A McKenna A Weber Viste J Fedorov E. Outcomes of clinical nurse specialist practice in the implementation of video monitoring at an academic medical center. Clin Nurse Spec. (2018) 32(2):90–6. 10.1097/NUR.0000000000000356 29419581

[39] Appenzeller YE Appelbaum PS Trachsel M. Ethical and practical issues in video surveillance of psychiatric units. Psychiatr Serv. (2020) 71(5):480–6. 10.1176/appi.ps.201900397 31847737

[40] Piotrowicz E Piepoli MF Jaarsma T Lambrinou E Coats AJS Schmid J-P. Telerehabilitation in heart failure patients: the evidence and the pitfalls. Int J Cardiol. (2016) 220:408–13. 10.1016/j.ijcard.2016.06.277 27390963

[41] Lara B Kottler J Olsen A Best A Conkright J Larimer K. Home monitoring programs for patients testing positive for SARS-CoV-2: an integrative literature review. Appl Clin Inform. (2021) 13(1):203–17. 10.1055/s-0042-1742370 PMC8850013 35172373

[42] van Uum RT Breteler M Hardeman H Van De Pol AC Smit K Boeijen JA. How Dutch initiatives to early discharge COVID-19 patients were organised during the pandemic: a scoping review. BMJ Open. (2025) 15(8):1–7. 10.1136/bmjopen-2024-097839 40858380 PMC12382530

[43] Yanes AF McElroy LM Abecassis ZA Holl J Woods D Ladner DP. Observation for assessment of clinician performance: a narrative review. BMJ Q Saf. (2016) 25(1):46–55. 10.1136/bmjqs-2015-004171 PMC13011875 26424762

[44] Kyriazakos S Pnevmatikakis A Kostopoulou K Ferrière L Thibaut K Giacobini E. Benchmarking the clinical outcomes of Healthentia SaMD in chronic disease management: a systematic literature review comparison. Front Public Health. (2024) 12:1–25. 10.3389/fpubh.2024.1488687 PMC11703908 39776481

[45] Ebad SA Alhashmi A Amara M Miled AB Saqib M. Artificial intelligence-based software as a medical device (AI-SaMD): a systematic review. Healthcare (Basel). (2025) 13(7). 10.3390/healthcare13070817 PMC11988595 40218113

[46] Gerke S Babic B Evgeniou T Cohen IG. The need for a system view to regulate artificial intelligence/machine learning-based software as medical device. NPJ Digit Med. (2020) 3:53. 10.1038/s41746-020-0262-2 32285013 PMC7138819

[47] Allen JA Reiter-Palmon R Jones KJ Sabalka L Ciagala K Meens A. Nurses’ experience implementing an automated video monitoring system to decrease the risk of patient falls during a global pandemic. Healthcare. (2023) 11(18):1–9. 10.3390/healthcare11182556 PMC10530789 37761753

[48] Lewis C Kearns T Shannon M. Beyond the bedside: the pivotal role of nursing clinical leadership in developing virtual ward care through telemedicine—a review of the evidence. J Clin Nurs. (2026):1–16. 10.1111/jocn.70413 PMC13629265 42323700

[49] Arksey H O'Malley L. Scoping studies: towards a methodological framework. Int J Soc Res Methodol. (2005) 8(1):19–32. 10.1080/1364557032000119616

[50] Levac D Colquhoun H O'Brien KK. Scoping studies: advancing the methodology. Implement Sci. (2010) 5(1):69. 10.1186/1748-5908-5-69 20854677 PMC2954944

[51] Tricco AC Lillie E Zarin W O'Brien KK Colquhoun H Levac D. PRISMA extension for scoping reviews (PRISMA-ScR): checklist and explanation. Ann Intern Med. (2018) 169(7):467–73. 10.7326/M18-0850 30178033

[52] Haddaway NR Collins AM Coughlin D Kirk S. The role of Google Scholar in evidence reviews and its applicability to grey literature searching. PLoS One. (2015) 10(9):e0138237. 10.1371/journal.pone.0138237 26379270 PMC4574933

[53] Hong QN Pluye P Fàbregues S Bartlett G Boardman F Cargo M. Mixed methods appraisal tool (MMAT), version 2018. Regist Copyright. (2018) 1148552(10):1–7. Available online at: https://bmjopen.bmj.com/content/bmjopen/11/2/e039246/DC3/embed/inline-supplementary-material-3.pdf

[54] Spano-Szekely L Winkler A Waters C Dealmeida S Brandt K Williamson M. Individualized fall prevention program in an acute care setting: an evidence-based practice improvement. J Nurs Care Qual. (2019) 34(2):127–32. 10.1097/NCQ.0000000000000344 30198949

[55] Davis J Carter-Templeton H. Augmenting an inpatient fall program with video observation. J Nurs Care Qual. (2021) 36(1):62–6. 10.1097/NCQ.0000000000000486 32282505

[56] Sosa MA Soares M Patel S Trujillo K Ashley D Smith E. The impact of adding a 2-way video monitoring system on falls and costs for high-risk inpatients. J Patient Saf. (2024) 20(3):186–91. 10.1097/PTS.0000000000001197 38345404

[57] Carayon P Wooldridge A Hoonakker P Hundt AS Kelly MM. SEIPS 3.0: human-centered design of the patient journey for patient safety. Appl Ergon. (2020) 84:103033. 10.1016/j.apergo.2019.103033 31987516 PMC7152782

[58] Greenhalgh T Wherton J Papoutsi C Lynch J Hughes G A'Court C. Beyond adoption: a new framework for theorizing and evaluating nonadoption, abandonment, and challenges to the scale-up, spread, and sustainability of health and care technologies. J Med Internet Res. (2017) 19(11):e367. 10.2196/jmir.8775 29092808 PMC5688245

[59] Dalkin SM Greenhalgh J Jones D Cunningham B Lhussier M. What’s in a mechanism? Development of a key concept in realist evaluation. Implement Sci. (2015) 10:49. 10.1186/s13012-015-0237-x 25885787 PMC4408605

[60] Greenhalgh T Wherton J Papoutsi C Lynch J Hughes G A’Court C. Analysing the role of complexity in explaining the fortunes of technology programmes: empirical application of the NASSS framework. BMC Med. (2018) 16(1):66. 10.1186/s12916-018-1050-6 PMC5950199 29754584

[61] Jeffries M Phipps DL Howard RL Avery AJ Rodgers S Ashcroft DM. Understanding the implementation and adoption of a technological intervention to improve medication safety in primary care: a realist evaluation. BMC Health Serv Res. (2017) 17(1):196. 10.1186/s12913-017-2131-5 28288634 PMC5348746

[62] Blijleven V Hoxha F Jaspers M. Workarounds in electronic health record systems and the revised sociotechnical electronic health record workaround analysis framework: scoping review. J Med Internet Res. (2022) 24(3):e33046. 10.2196/33046 35289752 PMC8965666

[63] Melnick ER Dyrbye LN Sinsky CA Trockel M West CP Nedelec L. The association between perceived electronic health record usability and professional burnout among US physicians. Mayo Clin Proc. (2020) 95(3):476–87. 10.1016/j.mayocp.2019.09.024 31735343

[64] Griffiths JL Saunders KRK Foye U Greenburgh A Regan C Cooper RE. The use and impact of surveillance-based technology initiatives in inpatient and acute mental health settings: a systematic review. BMC Med. (2024) 22(1):1–60. 10.1186/s12916-024-03673-9 39614242 PMC11605989

[65] Kristoffersson A Linden M. A systematic review on the use of wearable body sensors for health monitoring: a qualitative synthesis. Sensors. (2020) 20(5):1–21. 10.3390/s20051502 PMC7085653 32182907

[66] Paradis E Sutkin G. Beyond a good story: from Hawthorne effect to reactivity in health professions education research. Med Educ. (2017) 51(1):31–9. 10.1111/medu.13122 27580703

[67] Greenhalgh J Manzano A. Understanding ‘context’ in realist evaluation and synthesis. Int J Soc Res Methodol. (2022) 25(5):583–95. 10.1080/13645579.2021.1918484

[68] Skivington K Matthews L Simpson SA Craig P Baird J Blazeby JM. A new framework for developing and evaluating complex interventions: update of Medical Research Council guidance. Br Med J. (2021) 374:n2061. 10.1136/bmj.n2061 PMC8482308 34593508

[69] Chalke AM Leito GM Sidhu A McClelland J Agyemang S Luzingu JK. Practice and impact of using fall screening tools in emergency medicine for older adults: a scoping review. J Appl Gerontol. (2025) 44(10):1724–43. 10.1177/07334648251315279 39936381

[70] Venkatesh V Thong JYL Xu X. Unified theory of acceptance and use of technology: a synthesis and the road ahead. J Assoc Inf Syst. (2016) 17(5):328–76. 10.17705/1jais.00428

[71] Rouidi M Elouadi AE Hamdoune A Choujtani K Chati A. TAM-UTAUT and the acceptance of remote healthcare technologies by healthcare professionals: a systematic review. Inform Med Unlocked. (2022) 32:101008. 10.1016/j.imu.2022.101008

[72] Vandemeulebroucke T Dierckx de Casterlé B Gastmans C. The use of care robots in aged care: a systematic review of argument-based ethics literature. Arch Gerontol Geriatr. (2018) 74:15–25. 10.1016/j.archger.2017.08.014 28926749

[73] Hall A Brown Wilson C Stanmore E Todd C. Implementing monitoring technologies in care homes for people with dementia: a qualitative exploration using normalization process theory. Int J Nurs Stud. (2017) 72:60–70. 10.1016/j.ijnurstu.2017.04.008 28494333 PMC5490779

[74] Hall A Brown Wilson C Stanmore E Todd C. Moving beyond ‘safety’ versus ‘autonomy’: a qualitative exploration of the ethics of using monitoring technologies in long-term dementia care. BMC Geriatr. (2019) 19(1):145. 10.1186/s12877-019-1155-6 31126240 PMC6534927

[75] Dearing JW Cox JG. Diffusion of innovations theory, principles, and practice. Health Aff. (2018) 37(2):183–90. 10.1377/hlthaff.2017.1104 29401011

[76] May CR Cummings A Girling M Bracher M Mair FS May CM. Using normalization process theory in feasibility studies and process evaluations of complex healthcare interventions: a systematic review. Implement Sci. (2018) 13(1):80. 10.1186/s13012-018-0758-1 29879986 PMC5992634

[77] Damschroder LJ Reardon CM Widerquist MAO Lowery J. The updated consolidated framework for implementation research based on user feedback. Implement Sci. (2022) 17(1):75. 10.1186/s13012-022-01245-0 36309746 PMC9617234

[78] Cho OM Kim H Lee YW Cho I. Clinical alarms in intensive care units: perceived obstacles of alarm management and alarm fatigue in nurses. Healthc Inform Res. (2016) 22(1):46–53. 10.4258/hir.2016.22.1.46 26893950 PMC4756058

[79] Lyell D Coiera E. Automation bias and verification complexity: a systematic review. J Am Med Inform Assoc. (2017) 24(2):423–31. 10.1093/jamia/ocw105 27516495 PMC7651899

[80] Abbe JR O'Keeffe C. Continuous video monitoring: implementation strategies for safe patient care and identified best practices. J Nurs Care Qual. (2021) 36(2):137–42. 10.1097/ncq.0000000000000502 32658001 PMC7899219

[81] Waltz TJ Powell BJ Fernández ME Abadie B Damschroder LJ. Choosing implementation strategies to address contextual barriers: diversity in recommendations and future directions. Implement Sci. (2019) 14(1):42. 10.1186/s13012-019-0892-4 31036028 PMC6489173

[82] Curran GM Bauer M Mittman B Pyne JM Stetler C. Effectiveness-implementation hybrid designs: combining elements of clinical effectiveness and implementation research to enhance public health impact. Med Care. (2012) 50(3):217–26. 10.1097/MLR.0b013e3182408812 22310560 PMC3731143

